# Early innate immune events induced by prolonged cold ischemia exacerbate allograft vasculopathy

**DOI:** 10.1186/1749-8090-6-2

**Published:** 2011-01-06

**Authors:** Jennifer J Devitt, Chelsey L King, Timothy DG Lee, Camille L Hancock Friesen

**Affiliations:** 1 Department of Surgery, Dalhousie University, 5850 College Street, Halifax, B3H 1X5, Canada; 2 Department of Pathology, Dalhousie University, 5850 College Street, Halifax, B3H 1X5, Canada; 3 Department of Microbiology & Immunology, Dalhousie University, 5850 College Street, Halifax, B3H 1X5, Canada

## Abstract

**Background:**

Ischemia/reperfusion induced innate immune injury is inescapable in solid organ transplantation. Prolonged cold ischemia exacerbates the primary manifestation of late graft rejection, allograft vasculopathy (AV). The relationship between prolonged cold ischemia and late graft events is unclear and the subject of this study.

**Methods:**

Aortic interposition transplants were performed between fully disparate mice treated with CyclosporineA. Allografts were exposed to 20 min or 60 min of cold ischemia and harvested between 1 d-6 wk. Lesion size, smooth muscle cells (SMC), neutrophils (N∅), and CD8^+ ^T cells were quantified.

**Results:**

Early SMC loss was identical in both groups. When compared to 20 min cold ischemia, grafts exposed to 60 min exhibited greater early N∅ influx, greater SMC proliferation but fewer medial SMC at 1 wk and 2 wk. Subsequently, earlier and greater CD8^+ ^T cell infiltration were seen in the 60 min group with larger lesions at every time point.

**Conclusions:**

These data suggest that the larger neointimal lesions in grafts exposed to 60 min cold ischemia result from enhanced early innate immune events resulting in impaired SMC recovery and subsequent increased adaptive immune response.

## Introduction

Despite the introduction of mechanical assist devices to stabilize cardiac function, cardiac transplantation remains the primary treatment for end stage heart failure. Unfortunately, the long term (10 yr) survival of cardiac transplants has not improved in the last three decades and still hovers at approximately 50% in most centres [1]. The primary manifestation of late graft rejection in cardiac transplants is allograft vasculopathy (AV), observed predominantly in the large epicardial coronary vessels [2]. The etiology and pathology of AV are incompletely understood but it is characterized by vascular remodelling [2], including loss of medial smooth muscle cells (SMC) and the growth of a neointimal lesion. This lesion eventually narrows the vessel lumen and promotes thrombosis in the affected vessels. Although the etiology of AV remains to be completely elucidated, a number of studies have demonstrated the importance of both innate inflammatory events, as well as the adaptive immune response in the development of this disease [2,3].

We, and others, have demonstrated that T cell mediated immunity, particularly CD8^+ ^T cell immunity, plays a dominant role in the presence of calcineurin inhibitor (CNI) immunosuppression [4]. Humoral responses appear to play a role but this does not appear to be critical as B cell deficient animals develop similar AV lesions as their wild type littermates [5].

In contrast to the extensive studies that have investigated the adaptive arm of the immune response, there has been limited research into the early innate response and the suspected causative elements in this early response which activate later adaptive immunity. Since AV does not generally occur after transplantation into syngeneic recipients [6], or into immunodeficient animals (such as SCID or RAG knockout mice) [4], it is clear that the early innate inflammatory response does not itself directly cause AV but contributes to the later specific adaptive response.

Recently, focus has been brought to the potential contribution of the innate immune events to the development of AV [7,8]. This interest has been generated by evidence that some innate immune effectors can act as antigen presenting cells, thus linking them to adaptive immune mediated responses [9]. Indeed, it is reasonably well accepted that the pivotal event that initiates the complex process of graft rejection is the inevitable damage to the organ induced by ischemia reperfusion (IR) injury [10].

While efforts are made to limit cold ischemic times the acceptable ischemia duration is continually being challenged due to a shortage of donor organs. This is despite evidence from both clinical and experimental studies that prolonged cold ischemia (CI) has a negative impact on long term graft function and survival [1,11]. This situation is particularly acute for cardiac transplantation since poor cardiac graft outcomes have been convincingly demonstrated with ischemic times exceeding just 4 h [12].

There has been much speculation, but limited substantive evidence, regarding the manner by which prolonged CI results in worse acute and late graft outcomes. There is general agreement that prolonged CI likely mediates its effects very early post-transplantation and that these deleterious effects influence later events. Most studies examining early events post-transplantation have focused on endothelial cells, based on the assumption that endothelial cell dysfunction promotes neointimal lesion formation [13,14]. However, we [15] have demonstrated that medial events, rather than endothelial events, are pivotal in the induction of lesion formation. Further, we have recently shown that profound neutrophil (N∅) mediated SMC loss occurs by 1 d post-transplant [16] in an aortic interposition graft model of cardiac AV, under the cover of CNI immunosuppression. Donor SMC in the media recover from this loss by 4 wk post-transplant, only to be lost again as a result of the adaptive immune response. In this study, we investigate the effect of prolonged cold ischemia on these pivotal early innate events.

## Materials and Methods

### Animals

Male, 8-10 wk old C3H/HeJ (C3H; H-2^k^) mice were used as donors for 8-10 wk old male C57BL/6 (B6; H-2^b^) recipients. All mice were purchased from Jackson Laboratories (Bar Harbor, ME) and maintained in the Carlton Animal Care Facility, at the Sir Charles Tupper Medical Building in a pathogen free environment. Food and water were given *ad libitum*. All animal experimentation was undertaken in compliance with the guidelines of the Canadian Council on Animal Care, under an approved animal protocol.

### Aortic Transplantation

Abdominal aortic segments were transplanted as we have previously described [17]. Briefly, a section of abdominal aorta approximately 1 mm in length was harvested from the donor mouse and flushed with cold saline solution and stored in 4°C saline for either 20 min or 60 min before transfer into the recipient. The recipient infrarenal abdominal aorta was isolated. After proximal and distal clamping, the recipient aorta was transected and the donor aorta was interposed with proximal and distal end-to-end anastomoses using an 11-0 suture in an interrupted fashion. The clamps were removed and blood flow was confirmed by direct inspection.

### Immunosuppression

Cyclosporine (CyA; Sandimmune iv(tm)) was purchased from Queen Elizabeth II Hospital Pharmacy, Halifax, NS. Aortic allograft recipient mice were treated with 50 mg/kg/d CyA subcutaneously (in sterile saline) for the duration of the experiment.

### Histology

#### Paraffin embedded sections

*Paraffin embedded sections*: aortic grafts were harvested and flushed with heparinised saline and fixed in 10% formalin at 4°C for 16 h. Grafts were transferred to phosphate buffered saline (PBS) at 4°C for 1-2 h and then stored in 70% ethanol until processing. Paraffin sections (5 μm) were stained with haematoxylin and 0.5% eosin (H&E) for general histology. Stained sections were viewed under a light microscope.

#### Frozen sections

*Frozen sections*: aortic grafts were submerged in a cryopreservation solution of OCT:20% sucrose (2:1), flash frozen in liquid nitrogen and stored at -80°C until sectioning. Grafts were sectioned (8 μm) and slides were fixed in ice cold acetone for 2 min and allowed to dry before storage at -80°C.

### Immunohistochemistry

#### Neutrophils

Paraffin sections were deparaffinised in xylene, hydrated through an ethanol series, washed in PBS and digested with Proteinase K (Dako; Mississauga, ON). Endogenous peroxidase was quenched with a 15 min incubation in 3% hydrogen peroxide. Nonspecific blocking was performed with a 20 min incubation in Serum-Free Blocking solution (Dako; Mississauga, ON). The primary antibody used was rat-anti-mouse neutrophil (Cedarlane; Burlington, ON) 1:1500 in PBS + 5% blocking solution. A biotinylated rabbit-anti-mouse IgG H+L (Vector Labs Inc; Burlingame, CA), 1:400 in PBS + 5% blocking solution was used as the secondary antibody. Sections were washed with PBS and incubated with a peroxidase avidin/biotin complex kit (Vector Labs Inc; Burlingame, CA). One drop of prepared 3,3-diaminobenzidine (DAB; Dako; Mississauga, ON) in 1 ml of substrate buffer was used as the chromogen. Sections were counterstained with Mayer's Haematoxylin.

#### Ki-67

Paraffin sections were deparaffinised in xylene, hydrated through an ethanol series and washed in PBS. Antigen retrieval was performed by pressure cooking in sodium citrate buffer pH 6.0. Endogenous peroxidase was quenched with a 15 min incubation in 3% hydrogen peroxide. Non specific blocking was performed with a 60 min incubation in 5% normal rabbit serum. The primary antibody used was rat-anti-mouse Ki-67 (Dako; Mississauga, ON) 1:1000 in PBS + 5% rabbit serum. A biotinylated rabbit-anti-mouse IgG H+L (Vector Labs Inc; Burlingame, CA), 1:200 in PBS was used as the secondary antibody. Peroxidase development and counterstain were as above.

#### CD8^+ ^T cells

Frozen sections were removed from -80°C storage and allowed to come to room temperature for 30 min before being washed with PBS. Endogenous peroxidase was quenched with a 5 min incubation in 0.03% hydrogen peroxide. Nonspecific blocking was performed with a 60 min incubation in 10% normal goat serum. The primary antibody used was rat-anti-mouse CD8a (BD Pharmingen; Franklin Lakes, NJ) 1:25 in PBS + 5% goat serum. A biotinylated goat anti-rat (BD Pharmingen; Franklin Lakes, NJ), 1:100 in PBS + 5% goat serum was used as the secondary antibody. Peroxidase development and counterstain were as above.

### Digital analysis

Digital images of the H&E stained aortas were captured using a Zeiss Axiovert 200 and AxioCam camera (Carl Zeiss, Thornwood, NY). Intimal and medial areas of the aortic grafts were outlined and quantified using a digital image analysis program (Scion Image Software, Scion Corp., Frederick, MD).

### Statistics

Data are presented as mean ± SEM for each experimental group. All statistics were performed using GraphPad Prism^® ^(GraphPad software; San Diego CA, USA). Results were analyzed by one-way ANOVA and the Tukey-Kramer multiple comparisons test was used. Values of p < 0.05 were considered significant.

## Results

### Prolonged cold ischemia leads to earlier lesion formation

**Figure 1:**
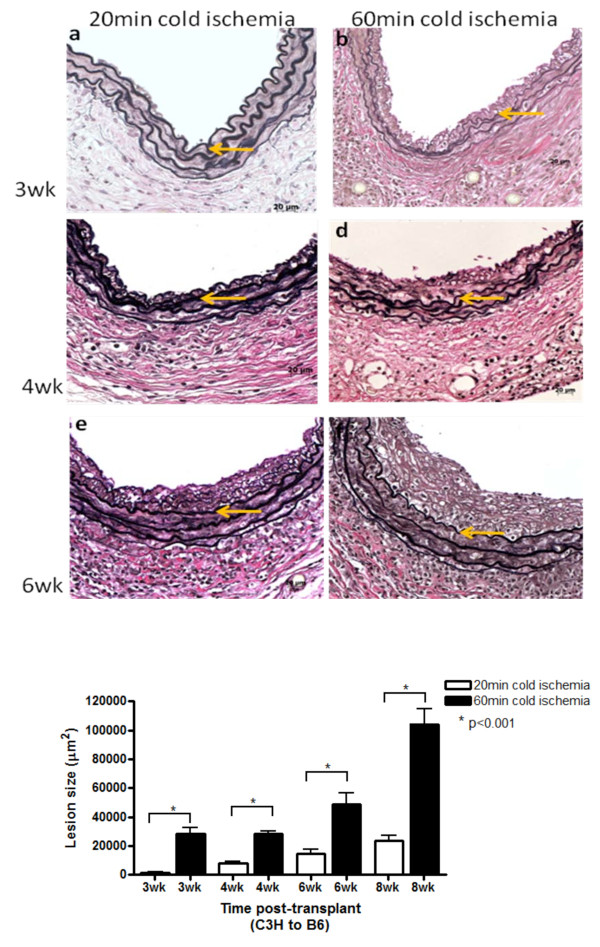
**Prolonged cold ischemia leads to earlier lesion formation**. Aortic allografts were exposed to 20 min cold ischemia (left panel) or 60 min cold ischemia (right panel) and harvested between 3 wk and 6 wk. Representative sections from elastin stained aortic allografts harvested at (a) and (b) 3 wk post-transplant; (c) and (d) 4 wk post-transplant; (e) and (f) 6 wk post-transplant; (g) lesion size as mean ± SEM. n = 4 animals per time point. Orange arrows point to internal elastic lamina.

Earlier studies conducted in the absence of CNI immunosuppression provide evidence that prolonged CI exacerbates AV, resulting in larger neointimal lesions [18]. To confirm this in the presence of clinically relevant levels of CNI immunosuppression, we followed the progression of lesion formation in transplanted murine aortic allografts which had been exposed to either 20 or 60 min of CI and harvested1-6 wk post-transplant. We used 50 mg/kg/d of CyA throughout the study to ablate acute rejection events. No lesions were seen in either the 20 min or 60 min ischemic groups before 2 wk post-transplant. By 3 wk, one out of four grafts exposed to 20 min of CI exhibited a small, focal lesion. In contrast, all (n = 4) of the grafts exposed to 60 min of CI had neointimal lesions and the mean lesion size in this group was markedly larger (p < 0.001; Figure 1). By 4 and 6 wk, lesions were evident in all grafts in both groups, but mean lesion size in the grafts exposed to 60 min cold ischemia was significantly larger (p < 0.001) at both 4 and 6 wk. These data confirm that under CNI immunosuppression 60 min CI leads to earlier and more robust neointimal lesions.

### Medial SMC recovery is hindered in grafts exposed to prolonged cold ischemia

**Figure 2:**
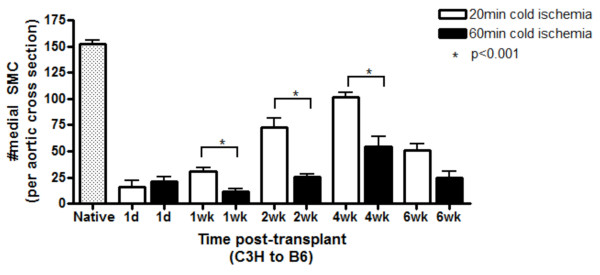
**Medial smooth muscle cell recovery is hindered in grafts exposed to prolonged cold ischemia**. Aortic allografts were exposed to 20 min cold ischemia (white bars) vs. 60 min cold ischemia (black bars) and harvested between 1 d-6 wk. A control group of native, non-transplanted aortic sections were removed and immediately processed for histology (stippled bar). Three randomly chosen sections were selected from each graft for digital image analysis to enumerate medial SMC numbers. Data shown here represents mean ± SEM. n = 4 animals per time point.

To explore the mechanisms responsible for the earlier and more robust development of neointimal lesions we examined the pathology associated with 60 min CI. Given that we have previously [19] demonstrated a link between medial SMC loss and lesion formation, we enumerated medial SMC at 1 d, 1 wk, 2 wk, 4 wk, and 6 wk post-transplant in both groups. Native, untransplanted C3H aortas were used as a control for baseline SMC counts. A dramatic and early loss of SMC numbers (> 90% versus baseline; p < 0.001) was observed at 1 d in both CI groups (Figure 2). There was no difference (p > 0.05) between the two groups in the magnitude of SMC loss. In both cases SMC loss was extensive.

In contrast to the equal medial SMC loss in both groups there were significant differences between the groups in timing and level of medial SMC recovery. In the 20 min CI group medial SMC numbers began to recover immediately, but in the 60 min CI group this recovery was significantly delayed and only started to be evident at 1 wk (p < 0.001; Figure 2). Whereas medial SMC counts in the 20 min CI group recovered to near normal levels by 4 wk, the 60 min CI group medial SMC counts remained significantly lower than baseline. After 4 wk, adaptive immune elements caused a second wave of medial SMC loss in both groups.

### Prolonged ischemia leads to greater early neutrophil influx

**Figure 3:**
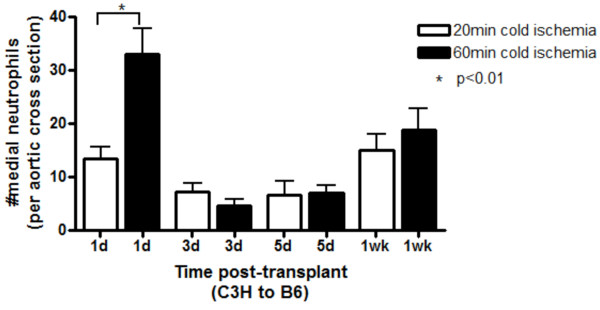
**Early neutrophil influx is greater in grafts exposed to prolonged cold ischemia**. Aortic allografts were exposed to 20 min or 60 min cold ischemia and harvested at 1 d, 3 d, 5 d and 1 wk post transplant. Three randomly chosen sections from each graft were stained for neutrophils. Neutrophil numbers in the media were enumerated. Data shown here represents mean ± SEM. n = 4 animals per time point.

Given their role in immediate innate inflammatory responses and tissue damage, and our previous evidence implicating N∅ as effectors in early AV events, we examined early N∅ influx in both CI groups. At 1 d post transplant, we found significantly (p < 0.01) higher medial N∅ infiltration into grafts exposed to 60 min CI than the 20 min group (Figure 3). At 3 and 5d post-transplant, the numbers of N∅ declined, with no significant difference between the two groups.

### Grafts exposed to prolonged cold ischemia show increased SMC proliferative activity

**Figure 4:**
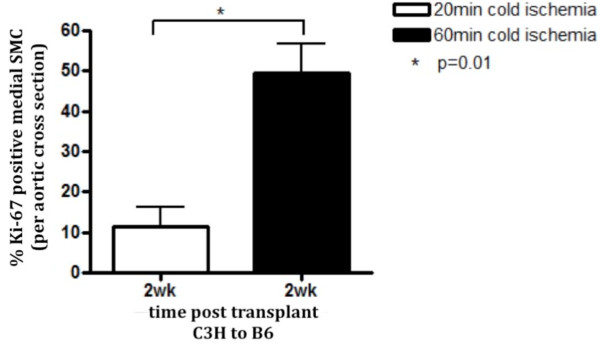
**Grafts exposed to prolonged cold ischemia have a higher percentage of proliferating medial SMC**. Aortic grafts exposed to 20 or 60 min of cold ischemia were harvested at 2 wk post transplant and stained using Ki-67 to assess proliferation. Ki-67 positive SMC were enumerated on three representative sections per graft. The percentage of proliferating SMC was calculated using the number of SMC enumerated on H&E sections. Data shown here represents mean ± SEM. n = 4 animals per time point.

As medial SMC in grafts exposed to 60 min CI showed impaired recovery, we assessed whether this was due to a lack of SMC proliferation in the media. Aortic grafts were harvested at 2 wk post-transplant and stained for Ki-67 to assess proliferation activity. The grafts exposed to 60 min cold ischemia displayed a significantly (p = 0.01) higher percentage of proliferating medial SMC than the grafts exposed to 20 min cold ischemia (Figure 4). This finding was consistent when we also examined the 4 wk time point (p = 0.03, data not shown) indicating that lack of proliferative activity was not responsible for the impaired recovery of SMC in the media of the grafts exposed to 60 min CI.

### CD8^+ ^T cells infiltrate the graft earlier when exposed to prolonged ischemia

**Figure 5:**
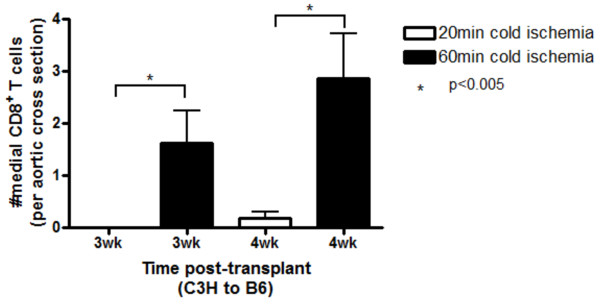
**CD8^+ ^T cells infiltrate earlier in grafts exposed to prolonged cold ischemia**. Aortic allografts exposed to 20 or 60 min cold ischemia were harvested at 3 and 4 wk post transplant and stained for CD8^+ ^T cells. Infiltrating CD8^+ ^T cells were enumerated in the media on three representative sections per graft and were analyzed using digital image analysis. Data shown here represents mean ± SEM. n = 4 animals per time point.

We have previously provided convincing evidence that, in this model, CD8^+ ^T cells are responsible for both SMC loss and lesion formation during the adaptive phase of the immune response [20]. Given that lesion formation was observed at earlier time points in the grafts exposed to 60 min CI, we investigated whether the earlier and more robust lesion formation correlates with earlier and more extensive CD8^+ ^T cell influx. Grafts were harvested at 3 and 4 wk post-transplant and stained for CD8^+ ^T cells. By 3 wk, only grafts exposed to 60 min CI exhibited CD8^+ ^T cell infiltration into the media (Figure 5). By 4 wk, both groups showed CD8^+ ^T cell influx, but the 60 min CI group showed markedly (p < 0.01) higher CD8^+ ^T cell counts.

## Discussion

It is well established that prolonging CI leads to poorer short and long term graft survival [12,18,21]. Despite this evidence, the lack of donor availability is driving longer ischemic times. We have previously shown that there is an early and profound loss of medial SMC post transplant in an aortic transplant model [16]. In this study, we examine the mechanisms responsible for the deleterious effect of prolonged CI on the development of AV. We used an aortic interposition model with clinically relevant levels of CNI immunosuppression (C2 levels approximating human levels) to ablate acute adaptive immune rejection events and reveal mechanistic detail of both early innate and late adaptive rejection responses. Clear evidence liking innate and adaptive immune responses can be invoked to develop a hypothesis by which prolonged CI could worsen AV by increasing the early innate inflammatory response and consequently augmenting the adaptive immune response [22]. We explored this hypothesis in our current set of experiments.

The two ischemic times we chose were designed to mimic the clinical situation, with 20 min in cold saline being the standard cold ischemic time in our laboratory and 60 min in cold saline being considered our upper limit for immediate graft function. Moreover, we were able to show pathologic differences between the two time points [16]. No doubt these time points could be adjusted upward by the use of preservation solution but they provided useful validated markers of long term graft function.

We first confirmed that 60 min CI would exacerbate vascular lesion formation in our model since this has not been previously demonstrated in models using CNI immunosuppression. Although it was not expected that CNI immunosuppression would dramatically alter early innate events (1 d-1 wk), the effects of CNI on the adaptive responses that are ultimately responsible for lesion formation are profound [4]. In this first section we examined lesion progression between 3-6 wk post-transplant. Our findings in this series of experiments confirmed that lesion formation was earlier and more robust in the 60 min CI group compared to the 20 min CI group.

IR damage to vascular tissue is widely held to be the pivotal event in the initiation of AV [22,23]. The specific target of that damage, however, is unclear and somewhat controversial. For example, Gohra and co-workers, using a rat aortic transplant model, demonstrated nearly complete endothelial denudation at 1 d post-transplant [13]. Similarly, Lai *et al*. showed endothelial destruction beginning at 1 d post-transplant in a rat heterotopic heart transplant model [14]. These data have suggested that early innate endothelial damage is the driving force behind the initiation of AV.

In contrast, more recent evidence has suggested that loss of medial SMC by late adaptive immune responses plays a dominant role in the development of AV [15]. For instance, we have demonstrated that CD8^+ ^T cells, Fas/FasL interactions, and IFN-γ expression, contribute to the late loss of medial SMC and the subsequent formation of the neointimal lesion [20]. However, the influence of early innate responses in the medial compartment early post transplant, on the subsequent development of AV has not been explored. Given the relationship between late SMC loss and AV, it would be plausible to link the early loss of SMC to this complex process. As such, we hypothesized that prolonged CI would lead to enhanced innate immune response to the graft, with impaired medial SMC viability, resulting in exacerbation of AV.

To explore this hypothesis we compared medial SMC numbers in grafts exposed to 20 vs. 60 min CI. There was a similar, immediate and dramatic loss of medial SMC at 1 d in both groups. Interestingly, by 1 wk, levels of medial SMC recovery demonstrated a remarkable divergence between the two ischemic groups. Medial SMC numbers recovered to near normal levels in the grafts exposed to 20 min CI, whereas the grafts exposed to 60 min CI showed markedly impaired medial SMC recovery.

To examine the mechanism behind the differences in SMC recovery between the two groups, we first examined N∅ influx. As predicted, there was substantial N∅ influx into the grafts by 1 d post transplant in both groups. A significantly increased N∅ infiltration was observed in the prolonged CI group at the 1 d time point, but this increased influx was not observed at any of the other time points examined (3 d, 5 d, 1 wk). In both groups, N∅ numbers followed the same pattern, in that at 1 wk there was a resurgence of medial N∅ infiltration which was similar in magnitude in both groups. The relationship of the increased N∅ influx at 1 d to later events, namely impaired SMC recovery, enhanced T cell influx and more robust lesion formation, is unclear. It may be that the enhanced early (1 d) N∅ influx amplifies the recruitment of other innate cells that exert downstream effects of the developing response. This could involve the elastin fragmentation in the media observed in this study and others.

To explore the nature of the impaired recovery we examined the possibility that medial SMC in the 60 min CI grafts demonstrate impaired ability to activate proliferation programs. Using a Ki-67 proliferation assay, we quantified the percentage of proliferating SMC within the media of the grafts. Surprisingly, the grafts exposed to 60 min CI had a significantly higher percentage of proliferating SMC than the 20 min group, suggesting the activation of a robust SMC repair mechanism among the SMC spared from the loss at 1 d. Despite this robust repair response in the 60 min CI grafts, it was not adequate to fully repopulate the media with SMC. In contrast, despite a lower percentage of proliferating SMC detected in the 20 min CI grafts, and a similar starting number of medial SMC at 1 d post transplant, the 20 min CI grafts did exhibit successful media repopulation. This strongly suggests that the proliferating donor medial SMC in the 60 min CI grafts are being killed by innate or adaptive immune elements. The mechanism of SMC death has yet to be elucidated. At 1 wk and 2 wk apoptotic cells (using TUNEL) were evident in the media but the numbers were similar between the 20 min and 60 min groups (data not shown). This leads us to believe that the loss of SMC may be due to necrosis but further studies are needed to fully rule out apoptosis.

The principle adaptive alloimmune response under CNI immunosuppression is mediated by CD8^+ ^T cells [4]. We have previously shown that CD8^+ ^T cells infiltrate the aortic grafts by 5 wk post transplant in the presence of CNI immunosuppression [19,20]. Our data here demonstrates that increased CI led to an earlier and more robust appearance of CD8^+ ^T cells into the media. Although the early and greater influx of cells of the adaptive response would account for the earlier and more robust lesion formation, they appear too late to account for the reduced recovery of SMC in the media. This implicates other cells of the innate response, for instance macrophages, in the impairment of recovery of medial SMC in the 60 min CI group and, potentially in the earlier initiation of the adaptive response. Further work in this area will be required to confirm this hypothesis.

## Conclusion

Prolonged CI leads to more intense early N∅ influx, extensive medial SMC damage, impaired recovery of medial SMC, earlier and more robust activation of adaptive immune responses and earlier and more robust neointimal lesion formation. Taken together, our data implicate the early influx of N∅ in the recruitment of other innate cells that limit the ability of the SMC to recover in the medial compartment. This leads to earlier and more robust expression of the adaptive response and subsequent lesion formation in AV.

## Competing interests

The authors declare that they have no competing interests.

## Authors' contributions

JD participated in the design, performed all allograft harvests, and helped draft the manuscript. CK performed the experimental procedures, with the exception of transplants and harvests, and helped draft the manuscript. TL and CHF conceived of the study, participated in its design, and helped draft the manuscript. All authors have read and approved the final manuscript.
